# Distinguishing dog-bite and rabies vulnerability in children to strengthen global elimination efforts

**DOI:** 10.1371/journal.pntd.0014680

**Published:** 2026-09-01

**Authors:** Harish Kumar Tiwari

**Affiliations:** 1 Jyoti and Bhupat Mehta School of Health Science and Technology, Indian Institute of Technology Guwahati, Guwahati, Assam, India; 2 DBT-Wellcome Trust India Alliance Intermediate Fellow, Hyderabad, Telangana, India; 3 JH CreIndia Foundation, Guwahati, Assam, India; Colorado State University, UNITED STATES OF AMERICA

## Abstract

Dog-mediated rabies remains a preventable cause of mortality in children, particularly in low- and middle-income countries where free-roaming dogs are common. While the WHO-led “Zero by 30” strategy emphasises mass dog vaccination and access to post-exposure prophylaxis (PEP), less attention has been paid to the behavioural and communication pathways that shape children’s vulnerability. This Viewpoint proposes a conceptual distinction between vulnerability to dog bites and vulnerability to rabies following exposure. Conflating these risks may contribute to diffuse messaging and missed prevention opportunities. This Viewpoint argues for a differentiated child-facing and adult-facing interventions, with schools positioned as adaptable platforms for integrating safe behaviour, disclosure, and timely care-seeking within existing systems. Aligning behavioural, educational, and biomedical components more explicitly may strengthen implementation of global rabies elimination strategies and reduce paediatric deaths.

Dog-mediated rabies is a neglected but entirely preventable cause of childhood mortality. More than one-third of rabies deaths occur in children younger than 15 years, mostly in low- and middle-income countries where free-roaming dogs (FRD) are ubiquitous [1]. In India alone, millions of children are exposed daily to potentially rabid encounters on streets, near schools and playgrounds, and around their homes. The heightened risk of dog bites in children is usually attributed to unpredictable gestures, spontaneous affection for dogs, short stature resulting in bites closer to the head, and limited awareness of rabies risk. While some countries have reported a decline in rabies incidence among children compared with older age groups, many rabies-endemic settings with a high incidence of dog bites continue to face persistent challenges in prevention and control [2]. The exposures may be provoked when dogs are pelted with stones, tail-pulled, or disturbed while feeding, or unprovoked, when FRD display territorial aggression, mating-season agitation, or rabid behaviour [3]. Global rabies elimination strategies under the ‘Zero by 30’ initiative prioritise mass dog vaccination, timely access to affordable PEP, and raising community awareness, including among children [4]. Yet, little attention has been paid to the distinct social and behavioural interventions that shape children’s vulnerabilities. It is argued that public health messaging should explicitly distinguish between vulnerability to dog bites and vulnerability to rabies to sharpen prevention strategies and bridge critical communication fault lines between children and adults with a duty of care.

Preventing the incidents of dog-bites in children requires pedagogical tools that build empathy, promote boundary recognition, and foster safe behaviours around dogs, while also teaching children what to do after a potential exposure. Evidence from schools indicates that structured education helps safer behaviour while interacting with dogs among children and discourages risky provocations. However, while reducing bites lowers exposure risk, it does not in itself prevent rabies deaths, which depend critically on adult-mediated actions following exposure. Rabies vulnerability begins only after an exposure has occurred and demands adult-mediated interventions such as improving communication to children to encourage disclosure of bite exposures, ensuring prompt and correct wound care, and securing time-critical access to rabies immunoglobulin and PEP [5]. To put it succinctly, many preventable dog-bite exposures can be reduced by empowering children to recognise risk, interact safely with dogs, and report exposures promptly. However, not all bites are behaviourally preventable. Rabies prevention ultimately depends on adult-mediated actions and system-level interventions, including mass dog vaccination, responsible dog population management, and timely access to post-exposure prophylaxis. Conflating these distinct vulnerabilities contributes to diffuse messaging and missed opportunities for rabies control.

An under-recognised barrier to timely prevention measures is children’s nondisclosure about dog encounters, often rooted in the fear of reprimand from parents or teachers who perceive FRDs as dirty or diseased. Behavioural and developmental research shows that children frequently conceal painful or frightening injuries when they anticipate reprimand, blame, or disbelief from authority figures, particularly in environments where animals are framed as dangerous or socially undesirable. Anticipatory guilt, fear of punishment, and uncertainty about the seriousness of an encounter can discourage disclosure of biting events even when injuries are severe [6]. At the same time, widespread misconceptions such as the belief that puppies cannot transmit rabies, or that scratches and licks are harmless reinforce children’s belief that disclosure is futile [7].

Qualitative evidence from educators, though limited, echoes this pattern and links nondisclosure with absent or delayed care and, ultimately, preventable mortality. Population-level data on the prevalence of nondisclosure remain sparse; however, the available findings suggest that communication barriers may contribute to delayed care-seeking. Timely wound washing and prompt initiation of PEP can transform a potentially fatal encounter into a survivable event, but only if the therapeutic window is not narrowed by nondisclosure. Reliable access to affordable PEP, rabies immunoglobulin where indicated, and functioning supply systems remain indispensable; however, these services can only prevent mortality when exposures are disclosed and care is sought in time. Communication barriers should therefore be considered a complementary implementation determinant rather than an alternative explanation for paediatric rabies deaths. Reframing dog-bites as distinct from rabies vulnerability, and addressing the associated family, school, and societal communication gaps, may represent an overlooked implementation gap.

An obvious corollary of this distinction is the need to move beyond the conventional collective approach to tackle dog-mediated rabies in children. At one end of the spectrum are child-facing strategies which should focus on age-appropriate curricula that nurture empathy for dogs, discourage provocation, and reinforce boundaries for safe interaction by instilling healthy behavioural and attitudinal practices. At the other end are the adult-facing strategies, aimed to resolve communication impasses through ‘ask-listen-act’ messaging. Parents, guardians and teachers should routinely ask the children about dog encounters, listen without blame or judgement, and act promptly by initiating wound washing and facilitating access to PEP, encouraging guilt-free disclosures of even seemingly minor encounters, enabling timely action. In practice, this could include teachers routinely asking children about recent dog encounters during school activities, parents responding calmly to reports of bites or scratches without assigning blame, and the use of age-appropriate storytelling, role-play, or visual materials that normalise disclosure and reinforce immediate wound washing and care-seeking. Community health workers and local volunteers can reinforce the same messages during outreach activities, allowing implementation to be adapted to local cultural and educational contexts. This dual-pronged approach aligns with stakeholder capacities as children can reduce avoidable exposure risks and report potentially dangerous encounters, while adults can act swiftly and decisively to prevent deaths from dog-mediated rabies. Within the WHO’s ‘Zero by 30’ global rabies elimination framework, which emphasises community awareness, bite management and access to PEP, a more explicit distinction between preventing dog bites and preventing rabies following exposure may strengthen implementation. Behavioural, educational and communication components are often bundled together, obscuring opportunities for more targeted and accountable interventions to prevent paediatric rabies deaths. The distinction between preventing dog bites and preventing rabies deaths in children can be mapped onto the core strategic interventions recommended under the ‘Zero by 30’ framework in Table 1, illustrating how existing priorities can be sharpened by explicitly aligning child-facing and adult-facing responsibilities.

**Table 1:** Aligning differentiated child-facing and adult-facing interventions for prevention of dog-mediated rabies within the WHO ‘Zero by 30’ framework.

Key implementation strategies of ‘Zero by 30’	Current emphasis	Gaps that affect implementation	Proposed school-linked addition (this Viewpoints)	Responsible stakeholder
Mass dog vaccination	Population level vaccination coverage of dogs to break the rabies transmission cycle	Lack of knowledge of importance of dog vaccination, Inaccessibility of dogs for immunisation, poor reporting of bites	School curricula that explain why dog vaccination matters, reinforce nonprovocative behaviour around dogs, and normalise reporting of bites from both owned and free-roaming dogs	Veterinary and municipal authorities (**delivery**); schools (**reinforcement**); children (**safe behaviour**)
Access to PEP	Timely availability of vaccines and rabies immunoglobulin through health facilities	Delayed disclosure or recognition of exposure; uneven access to affordable PEP and rabies immunoglobulin; delays in reaching appropriate care	School-based messaging that emphasises the urgency of disclosure, wound washing, and early care-seeking after a bite or scratch, or saliva exposure to broken skin or mucous membranes	Parents/guardians/teachers and health system (**action**); schools (**prompting**); children (**reporting**)
Community awareness and education	General population-level rabies awareness campaigns	Tailor made communication tools, especially for various age group children; Identifying important communication flow channels such as teachers -parents	Age-appropriate school curricula that distinguish child-facing dog-bite risk reduction from adult-mediated rabies prevention, including parent-teacher communication prompts.	Schools and educators (**delivery**); parents (**response**); children (**engagement**)
Integrated bite case management (IBCM)	Surveillance, reporting, and coordination between animal and human health sectors	Identifying schools at point of gathering information; School systems as facilitators for timely reporting	Schools as sentinel sites for early reporting of dog-bite incidents, facilitating faster referral and documentation through adult-led systems	Health and veterinary services (**management**); schools (**notification**); parents (**follow-up**)
Dog population management	Sterilisation, vaccination, waste management, urban planning, and sheltering	Responsible dog ownership, dog-utility, and animal welfare	Explicit messaging within school programmes against abandoning litters on streets, that dog population control, sheltering and environmental management remain adult and municipal responsibilities	Municipal authorities and adult systems (**implementation**); Schools (**messaging**)
Behaviour towards dogs	Largely undifferentiated messaging to ‘the community’	Specific definitions of safe behaviour, especially among children	Stratified communication: child-facing messages for safe interaction and disclosure; adult-facing messages focused on blame-free response, rapid care-seeking and adherence to PEP	Children (safe **behaviour where feasible and reporting**); adults and public health systems (**decision making, care and prevention**)

The delivery of these interventions requires multiple complementary platforms and stakeholders, of which schools represent one important entry point rather than the only mechanism for implementation. They represent an overlapping zone where both the strategies converge through curricular integration, parent-teacher exchanges, and counselling prompts that normalise disclosure and reduce stigma can be effectively planned and delivered at the level of educators. However, in settings where school attendance is limited or inconsistent, including children from resource deprived social groups, and transient or migrant populations, the complementary community-based approaches delivered through health workers, civil society organisations, and local networks become an important pathway ensuring equitable reach. Evidence shows that teachers are willing partners in this strategy when given the right tools and support [8]. By embedding messages that link compassion and affection with safety without vilifying FRD, school pedagogy can shape healthier attitudes in children. Such early education may also contribute to longer-term attitudes of responsible and respectful coexistence with animals as children transition into adulthood. A dialogue with parents can accelerate the destigmatisation of reporting, improve perceptions about dogs, and reinforce adherence to PEP.

Importantly, the integration of schools into rabies prevention is not merely aspirational but has already been implemented, formally or informally, in several rabies-endemic settings. In the Philippines, school-based information and education campaigns have been embedded within broader rabies control efforts, demonstrating measurable gains in students’ knowledge of rabies transmission, bite prevention, and appropriate post-exposure actions [9]. These initiatives have leveraged schools as trusted institutional platforms to normalise early disclosure of dog bites and reinforce timely care-seeking, while remaining anchored to adult-led systems for vaccination, clinical management, and dog population control [10,11]. Parallel evidence from school-based dog-bite prevention programmes in other settings further indicates that structured education can improve children’s knowledge and safer interactions with dogs [12]. Collectively, these experiences suggest that schools can function as effective intermediaries between children and adult systems of care, enhancing the reach and coherence of rabies prevention strategies without shifting responsibility away from parents, health services, or municipal authorities. Synthesising these implementation experiences strengthens the case for more explicit and differentiated school-linked communication within the ‘Zero by 30’ framework.

Implementation need not depend on resource-intensive programmes. Practical tools may include age-appropriate visual materials, role-play, simple bite-reporting prompts, parent-teacher communication aids, and culturally adapted counselling messages that can be incorporated into existing school and community health activities. In resource-constrained settings, such interventions could be embedded within ongoing rabies control, school health, municipal, or community outreach programmes, with additional support drawn from local government, civil-society organisations, and development partners working in child health, One Health, or neglected tropical diseases.

Metrics for the child-facing strategy could include reductions in dog bites, increased reporting of animal encounters, and documented safe behaviours around dogs. For the adult-facing strategy, indicators could include the time from bite to facility reporting, documentation of wound washing, timeliness of PEP initiation, and adherence to vaccination schedules. Developing novel, cost-effective communication interventions could substantially reduce child rabies deaths, while qualitative studies with children, parents, and teachers can refine messages and reduce stigma.

A child’s life should not depend on fear of reprimand following interaction with a dog. In rabies-endemic settings, the difference between survival and tragedy is often just a conversation between a child willing to tell, and an adult ready to act. It will take more than human or canine vaccines, guidelines, or trials for dog population management to end paediatric rabies deaths. It is time to identify and separate the two problems that have long been treated as one: reducing avoidable dog-bite exposures, where children can be empowered through age-appropriate education, and preventing rabies, which remains the responsibility of adults and public health systems. The social and familial stigmas that silence children must be erased by breaking communication barriers, aligning messages to the right audiences, and embedding dialogue within schools and communities. These actions, alongside mass dog vaccination and integrated bite case management, are essential to turn the elusive promise of ‘Zero by 30’ into a reality.

## Declaration of generative AI and AI-assisted technologies in the manuscript preparation process

During the preparation of this work, the author used **Grammarly** in order to improve readability and grammar. After using this tool/service, the author(s) reviewed and edited the content as needed and take(s) full responsibility for the content of the published article.
